# Effectiveness comparison of indocyanine green retention test with the cirrhotic severity scoring in evaluating the pathological severity of liver cirrhosis in patients with hepatocellular carcinoma and Child-Pugh grade A liver function

**DOI:** 10.1186/s12957-020-01854-3

**Published:** 2020-04-23

**Authors:** Jin Gu, Erlei Zhang, Binyong Liang, Zunyi Zhang, Xiaoping Chen, Zhiyong Huang

**Affiliations:** Hepatic Surgery Center, Tongji Hospital, Tongji Medical College, Huazhong University of Science and Technology, 1095 Jie Fang Da Dao, Wuhan, China

**Keywords:** Hepatocellular carcinoma, Liver cirrhosis, Laennec staging system, Indocyanine green retention test, Cirrhotic severity scoring, Posthepatectomy liver failure, Mortality

## Abstract

**Background:**

Evaluating cirrhotic severity is essential for individualizing surgical modalities for patients with hepatocellular carcinoma (HCC). Our previous study proposed a non-invasive method named cirrhotic severity scoring (CSS) to stage liver cirrhosis. Indocyanine green retention rate at 15 min (ICG-R15) has been widely used for the preoperative evaluation of hepatic functional reserve; however, whether ICG-R15 is well correlated with cirrhotic severity, and especially whether comparable with CSS in predicting cirrhotic severity in HCC patients with Child-Pugh grade A liver function remains unknown.

**Methods:**

Overall, 510 HCC patients with Child-Pugh grade A liver function undergoing hepatectomy between January 2011 and December 2014 were retrospectively studied. Cirrhotic severity was pathologically assessed using the Laennec staging system. The correlations between ICG-R15, CSS, and cirrhotic severity were analyzed. Furthermore, the performance of ICG-R15 and CSS in predicting posthepatectomy liver failure (PHLF) and 90-day mortality was compared.

**Results:**

Patients with no, mild, moderate, and severe cirrhosis accounted for 15.9%, 29.2%, 35.9%, and 19.0%, respectively, in the entire cohort. ICG-R15 was found to be less than 10% in 100%, 93.3%, 86.3%, and 70.1% of the patients with no, mild, moderate, and severe cirrhosis, respectively. There was only a weak correlation between ICG-R15 and the pathological severity of liver cirrhosis (*r* = 0.325; *P* < 0.001). However, CSS showed a strong correlation with the pathological severity of liver cirrhosis (*r* = 0.788; *P* < 0.001). For those with ICG-R15 in the normal range, the accuracy of CSS in diagnosing no/mild, moderate, and severe cirrhosis was 89.1%, 72.8%, and 72.1%, respectively. In addition, CSS was superior to ICG-R15 in predicting PHLF and 90-day mortality.

**Conclusions:**

CSS was more useful than ICG-R15 in the preoperative assessment of cirrhotic severity in HCC patients with Child-Pugh grade A liver function. More studies are needed to further validate CSS in patients with different Child-Pugh grades.

## Introduction

Hepatocellular carcinoma (HCC) is the sixth most common malignancy and the fourth leading cause of cancer-related death in the world [1]. Hepatectomy remains the mainstay curative option for HCC patients [2]. However, in China, more than 80% of patients develop HCC in the background of cirrhosis [3]. Posthepatectomy liver failure (PHLF) resulting from the insufficient residual liver function is one of the most serious complications for HCC patients undergoing hepatic resection [4, 5], and the incidence of PHLF is significantly associated with cirrhotic severity [6]. Therefore, preoperative evaluation of cirrhotic severity is of significant importance before making a surgical treatment strategy.

Indocyanine green (ICG) is a water-soluble anionic compound that mainly binds to plasma proteins after intravenous administration. It is selectively taken up by hepatocytes and excreted unchanged into the bile. It is not metabolized and does not enter the enterohepatic circulation [7]. The clearance of ICG relies on the function of hepatocytes, biliary excretion, and liver blood flow [8, 9]. ICG retention rate at 15 min (ICG-R15) is a widely used clinical parameter calculated from liver clearance of ICG. Many studies have indicated that ICG-R15 could serve as a quantitative parameter in assessing liver functional reserve [10, 11]. However, whether ICG-R15 is effective in evaluating cirrhotic severity in HCC patients with Child-Pugh grade A liver function has never been elucidated.

In the assessment of cirrhotic severity, liver biopsy is still regarded as the reference standard. Nevertheless, it is an invasive procedure, and its results are influenced by several factors, such as inter-observer variability and sampling errors [12, 13]. Thus, liver biopsy is not recommended to be performed in the preoperative evaluation of cirrhotic severity. The cirrhotic severity scoring (CSS) was previously developed by our team as a non-invasive method to stage liver cirrhosis in HCC patients [14]. It relies on the patient’s portal vein diameter, splenic thickness, platelet count, and varicosity, which are routinely measured and available for virtually all patients with HCC scheduled for hepatectomy. This method has exhibited a high degree of diagnostic accuracy in predicting the pathological severity of liver cirrhosis; however, whether CSS is superior to ICG-R15 in assessing cirrhotic severity remains unexplored.

The present study aimed to investigate the effectiveness of ICG-R15 in the preoperative evaluation of cirrhotic severity in HCC patients with Child-Pugh grade A liver function and compare its diagnostic performance with that of CSS. Furthermore, the performance of ICG-R15 and CSS in predicting PHLF and 90-day mortality was compared.

## Patients and methods

### Patients

This study was conducted in accordance with the standards of the Declaration of Helsinki and approved by the medical ethics committee of Tongji Hospital, Huazhong University of Science and Technology, China. Written informed consent for hepatectomy and further research was obtained from all patients. A total of 510 HCC patients with Child-Pugh grade A liver function who underwent hepatic resection at the Hepatic Surgery Center of Tongji Hospital between January 2011 and December 2014 were retrospectively analyzed. All patients received routine blood tests, chest X-ray, abdominal ultrasonography, upper gastrointestinal endoscopy, computed tomography, and ICG-R15 for preoperative assessment. Clinicopathological records of all patients were obtained from the computerized database maintained by Tongji Hospital. The grades of esophageal varices were classified as follows: mild, straight varices not disappearing with insufflations; moderate, enlarged tortuous, occupying less than 1/3 of the lumen; and severe, coil-shaped, occupying more than 1/3 of the lumen [15]. Major resection was defined as resection of 3 or more Couinaud liver segments [16]. PHLF was defined by an increased international normalized ratio and concomitant hyperbilirubinaemia on or after postoperative day 5 as proposed by the International Study Group of Liver Surgery [4]. Postoperative mortality was defined as death within 90 days after hepatectomy.

### ICG retention test

ICG retention test was carried out by injecting a dose of 0.5 mg/kg of ICG rapidly via a peripheral vein of the forearm. A nasal mucosa probe was employed with the purpose of monitoring changes in ICG concentrations. The value of ICG-R15 was calculated by a Pulse Dye Densito-Graph Analyzer (DDG-3300 K, Nihon Kohden, Tokyo, Japan).

### Pathological assessment of cirrhotic severity

The pathological severity of liver cirrhosis in the non-tumorous tissue of the resected liver specimens was evaluated by two experienced pathologists who were blinded to the clinical information according to the Laennec staging system. Cirrhotic severity was staged as follows: no cirrhosis (combination of F0-F3); mild cirrhosis (F4A, marked septation with rounded contours); moderate cirrhosis (F4B, at least 2 broad septa); and severe cirrhosis (F4C, at least 1 very broad septum or many minute nodules) [17].

### Cirrhotic severity scoring (CSS) for evaluating cirrhotic severity

The calculation of CSS was based on the portal vein diameter, splenic thickness, platelet count, and varicosity as described in Table 1 [14].

**Table 1:** Assessment of cirrhotic severity according to the cirrhotic severity scoring

Variables	Score		
	0	1	2
Portal vein diameter (cm)	< 1.2	1.2-1.4	> 1.4
Splenic thickness (cm)	< 4.0	4.0-5.0	> 5.0
Platelet count (10^9^/L)	≥ 100	70-100	< 70
Varicosity	No	Mild	Moderate/severe
	No/mild cirrhosis	Moderate cirrhosis	Severe cirrhosis
CSS	0-1	2-3	≥ 4

*CSS* cirrhotic severity scoring

### Statistical analysis

Continuous variables were expressed as mean ± standard deviation. Categorical variables were expressed as numbers and percentages and compared using Fisher’s exact test. Spearman correlation coefficients were used to analyze the correlations between ICG-R15, CSS, and cirrhotic severity, and correlation coefficients were compared using Fisher *Z* test. The predictive ability of ICG-R15 and CSS was assessed using receiver operating characteristic (ROC) curves. Areas under the ROC curves (AUCs) were compared using the Delong method [18]. *P* < 0.05 was considered statistically significant. Statistical analyses were performed using the SPSS version 20.0 for Windows (SPSS Inc, Chicago, IL, USA) and Medcalc version 18.2.1 (MedCalc Software bvba, Mariakerke, Belgium).

## Results

### Patients’ characteristics

**Fig. 1:**
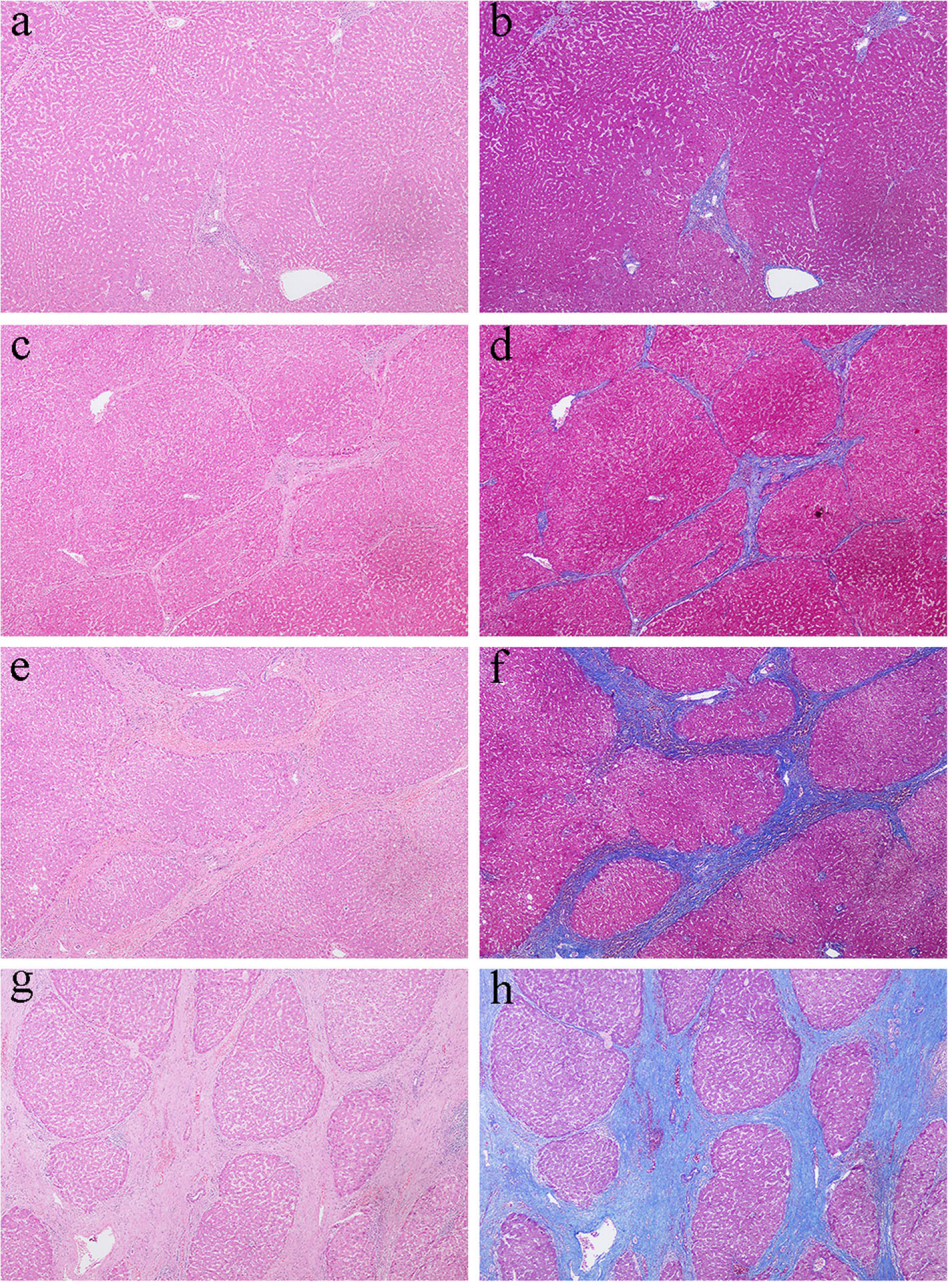
Pathological sub-classification of liver cirrhosis according to the Laennec staging system. **a**, **b** Show no cirrhosis (combination of F0-F3) (H&E and Masson trichrome stain, respectively, × 40). **c**, **d** Show mild cirrhosis (F4A) (H&E and Masson trichrome stain, respectively, × 40). **e**, **f** Show moderate cirrhosis (F4B) (H&E and Masson trichrome stain, respectively, × 40). **g**, **h** Show severe cirrhosis (F4C) (H&E and Masson trichrome stain, respectively, × 40). H&E, hematoxylin-eosin

The characteristics of all patients are summarized in Table 2. There were 449 men and 61 women, and the mean age was 50.1 ± 11.2 years. Hepatitis B was the main etiology (89.6%) followed by hepatitis C (2.0%). The mean value of ICG-R15 was 5.7 ± 4.6%. The mean tumor diameter was 6.6 ± 3.7 cm (range, 1.0-20.0 cm). Four hundred and forty-seven patients (87.6%) had a single tumor, and 63 (12.4%) had 2 or more tumors. Surgical resections included 92 major resections and 418 minor resections. Based on the Laennec staging system, 81 patients (15.9%) were without cirrhosis (F0-F3), whereas 149 (29.2%) were diagnosed with mild cirrhosis (F4A), 183 (35.9%) with moderate cirrhosis (F4B), and 97 (19.0%) with severe cirrhosis (F4C). Representative figures of the corresponding Laennec stage are exhibited in Fig. 1.

**Table 2:** Baseline characteristics and postoperative short-term outcomes of the study population

Variables	Patients (*n* = 510)
Age (years)	50.1 ± 11.2
Sex, *n* (%)	
Men	449 (88.0%)
Women	61 (12.0%)
Etiology, *n* (%)	
Hepatitis B	457 (89.6%)
Hepatitis C	10 (2.0%)
Other	43 (8.4%)
ALT (U/L)	36.5 ± 24.6
AST (U/L)	39.2 ± 21.2
Albumin (g/L)	39.7 ± 4.5
Total bilirubin (μmol/L)	13.4 ± 5.2
Creatinine (μmol/L)	70.5 ± 15.2
Platelet count (10^9^/L)	143.6 ± 72.5
INR	1.1 ± 0.1
PT (s)	13.1 ± 1.6
ICG-R15 (%)	5.7 ± 4.6
Tumor size (cm)	6.6 ± 3.7
Tumor number, *n* (%)	
1	447 (87.6%)
≥ 2	63 (12.4%)
Portal vein diameter (cm)	1.2 ± 0.2
Splenic thickness (cm)	4.0 ± 0.8
Varicosity, *n* (%)	
No	437 (85.7%)
Mild	43 (8.4%)
Moderate	13 (2.5%)
Severe	17 (3.3%)
CSS, *n* (%)	
No/mild cirrhosis	246 (48.2%)
Moderate cirrhosis	169 (33.1%)
Severe cirrhosis	95 (18.6%)
Extent of hepatectomy, *n* (%)	
Major resection	92 (18.0%)
Minor resection	418 (82.0%)
Intraoperative blood loss (ml)	379.6 ± 378.9
Intraoperative RBC transfusion, *n* (%)	88 (17.3%)
Pathological stage, *n* (%)	
F0-F3	81 (15.9%)
F4A	149 (29.2%)
F4B	183 (35.9%)
F4C	97 (19.0%)
PHLF, *n* (%)	59 (11.6%)
90-day mortality, *n* (%)	9 (1.8%)

Data are expressed either as mean ± standard deviation for continuous variables or as number of patients with percentages in parentheses for categorical variables

*ALT* alanine aminotransferase; *AST* aspartate aminotransferase; *INR* international normalized ratio; *PT* prothrombin time; *ICG-R15* indocyanine green retention rate at 15 min; *CSS* cirrhotic severity scoring; *RBC* red blood cell; *PHLF* posthepatectomy liver failure

### Relationship between ICG-R15 and the Laennec stage

**Fig. 2:**
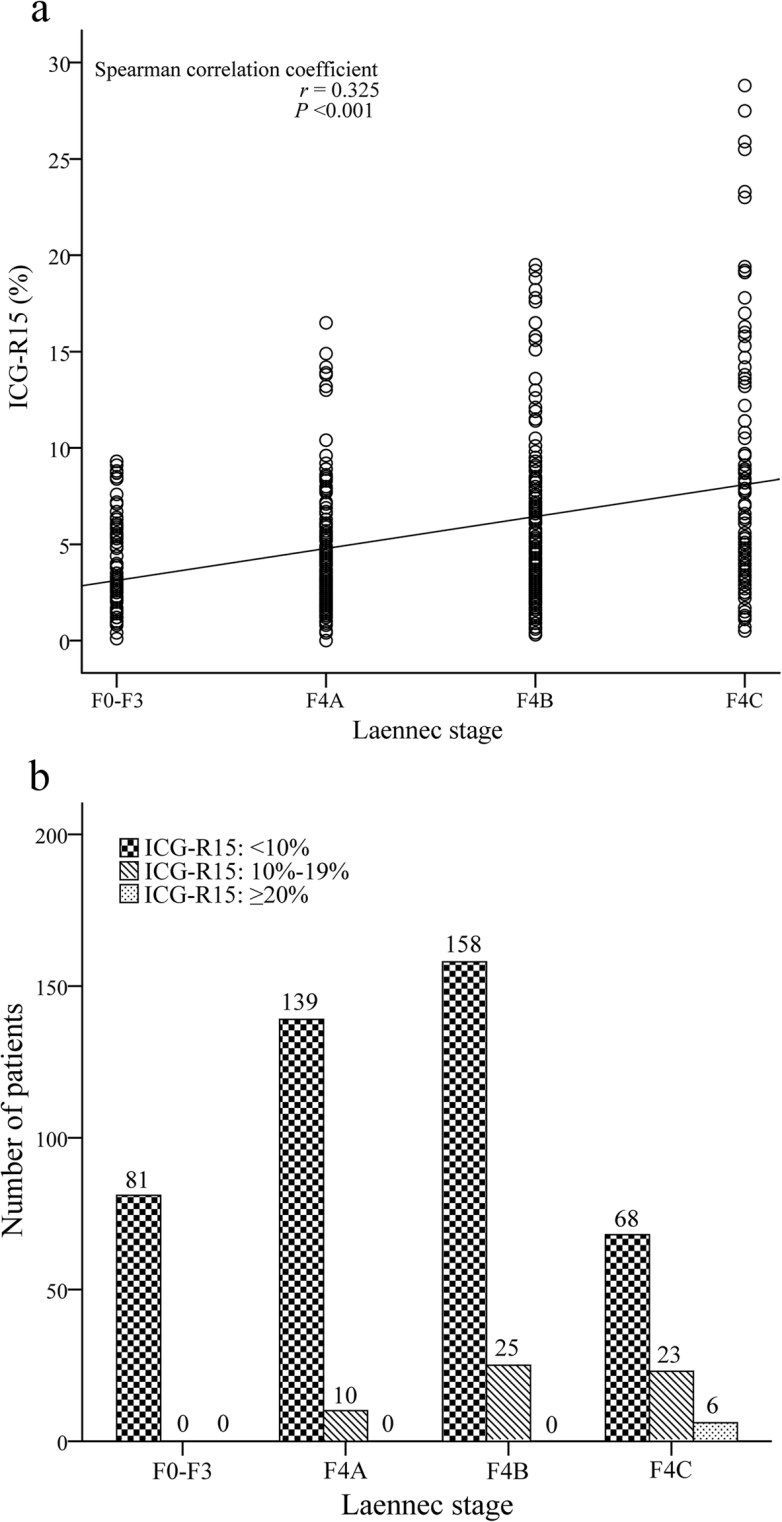
**a** Correlation between ICG-R15 and the Laennec stage. **b** The bar chart indicates the pattern of ICG-R15 distribution in the corresponding pathological stage of liver cirrhosis. ICG-R15, indocyanine green retention rate at 15 min

Although a positive correlation was observed between ICG-R15 and the Laennec stage in the entire cohort, the Spearman correlation coefficient was only 0.325 (*P* < 0.001; Fig. 2a), which reflected a very weak relationship. The number of patients with different levels of ICG-R15 in the corresponding pathological stage of liver cirrhosis is shown in Fig. 2b. In the no cirrhosis (F0-F3) group, all patients (100%) had ICG-R15 values below 10%. In the mild cirrhosis (F4A) group, 139 patients (93.3%) had ICG-R15 values below 10%, and 10 (6.7%) had ICG-R15 values between 10% and 19%. In the moderate cirrhosis (F4B) group, 158 patients (86.3%) had ICG-R15 values below 10%, and 25 (13.7%) had ICG-R15 values between 10 and 19%. In the severe cirrhosis (F4C) group, 68 patients (70.1%) had ICG-R15 values below 10%, 23 (23.7%) had ICG-R15 values between 10 and 19%, and 6 (6.2%) had ICG-R15 values ≥ 20%.

### CSS is significantly superior to ICG-R15 in evaluating cirrhotic severity

**Fig. 3:**
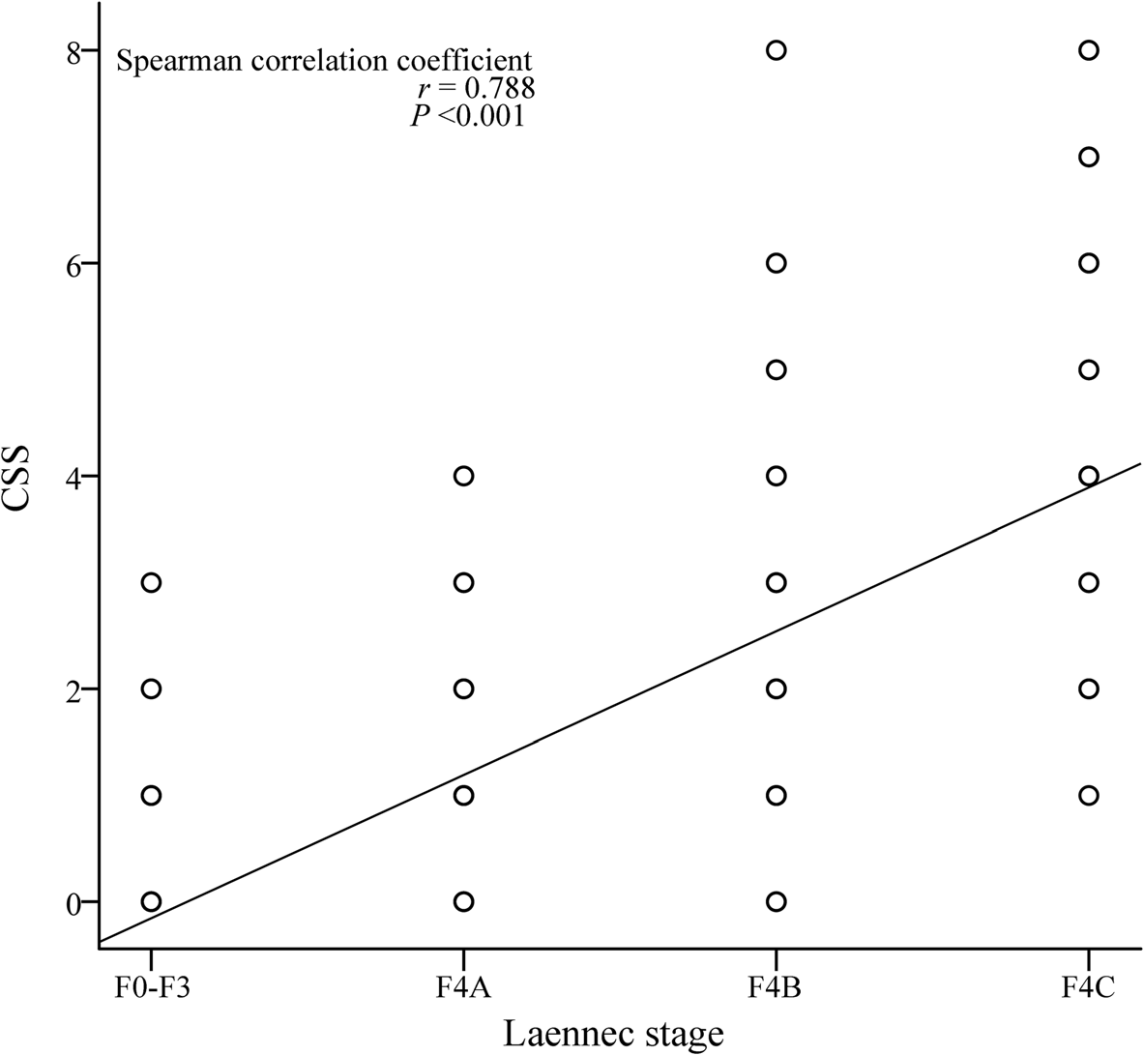
Correlation between CSS and the Laennec stage. CSS, cirrhotic severity scoring

**Fig. 4:**
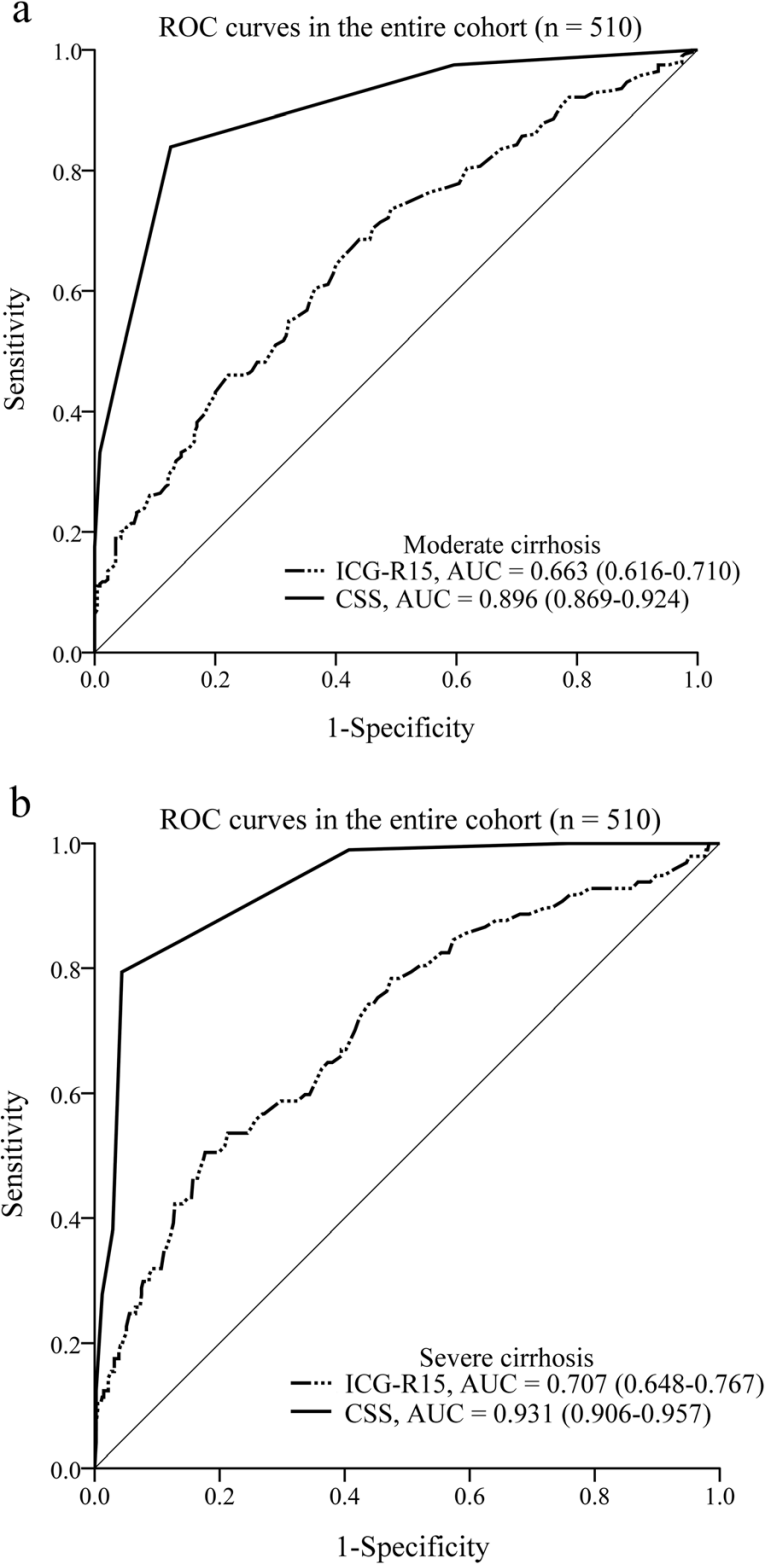
Receiver operating characteristic (ROC) curves and the corresponding areas under the ROC curves (AUCs) of ICG-R15 and CSS in the diagnosis of moderate (**a**) and severe cirrhosis (**b**) in the entire cohort. ICG-R15, indocyanine green retention rate at 15 min; CSS, cirrhotic severity scoring

CSS showed a strong correlation with the Laennec stage (*r* = 0.788; *P* < 0.001; Fig. 3). The Spearman correlation coefficient for CSS was significantly higher than that for ICG-R15 (*P* < 0.001). The diagnostic performance of ICG-R15 and CSS in the entire cohort is shown in Fig. 4. ROC analysis revealed that CSS exhibited a better performance than ICG-R15 in the diagnosis of moderate (AUC = 0.896 vs 0.663; *P* < 0.001) and severe cirrhosis (AUC = 0.931 vs 0.707; *P* < 0.001).

### Diagnostic accuracy of CSS in discriminating cirrhotic severity in patients with a normal ICG-R15 value

We further assessed the discriminative ability of CSS for cirrhotic severity in 446 patients with ICG-R15 in the normal range. The proportions of patients with Laennec stage F0-F3, F4A, F4B, and F4C were 18.2%, 31.2%, 35.4%, and 15.2%, respectively. According to the CSS, 233 patients (52.2%) had no/mild cirrhosis, 155 (34.8%) had moderate cirrhosis, and 58 (13.0%) had severe cirrhosis. The CSS was validated by comparison with the pathologically confirmed cirrhotic stage (Laennec stage). CSS was correct in 89.1% of patients with Laennec stage F0-F4A (*n* = 220), 72.8% with F4B (*n* = 158), and 72.1% with F4C (*n* = 68). Furthermore, ROC analysis showed that the AUCs of CSS in the diagnosis of moderate and severe cirrhosis in patients with a normal ICG-R15 value were 0.897 and 0.929, respectively.

### Performance of ICG-R15 and CSS in predicting PHLF and mortality

**Fig. 5:**
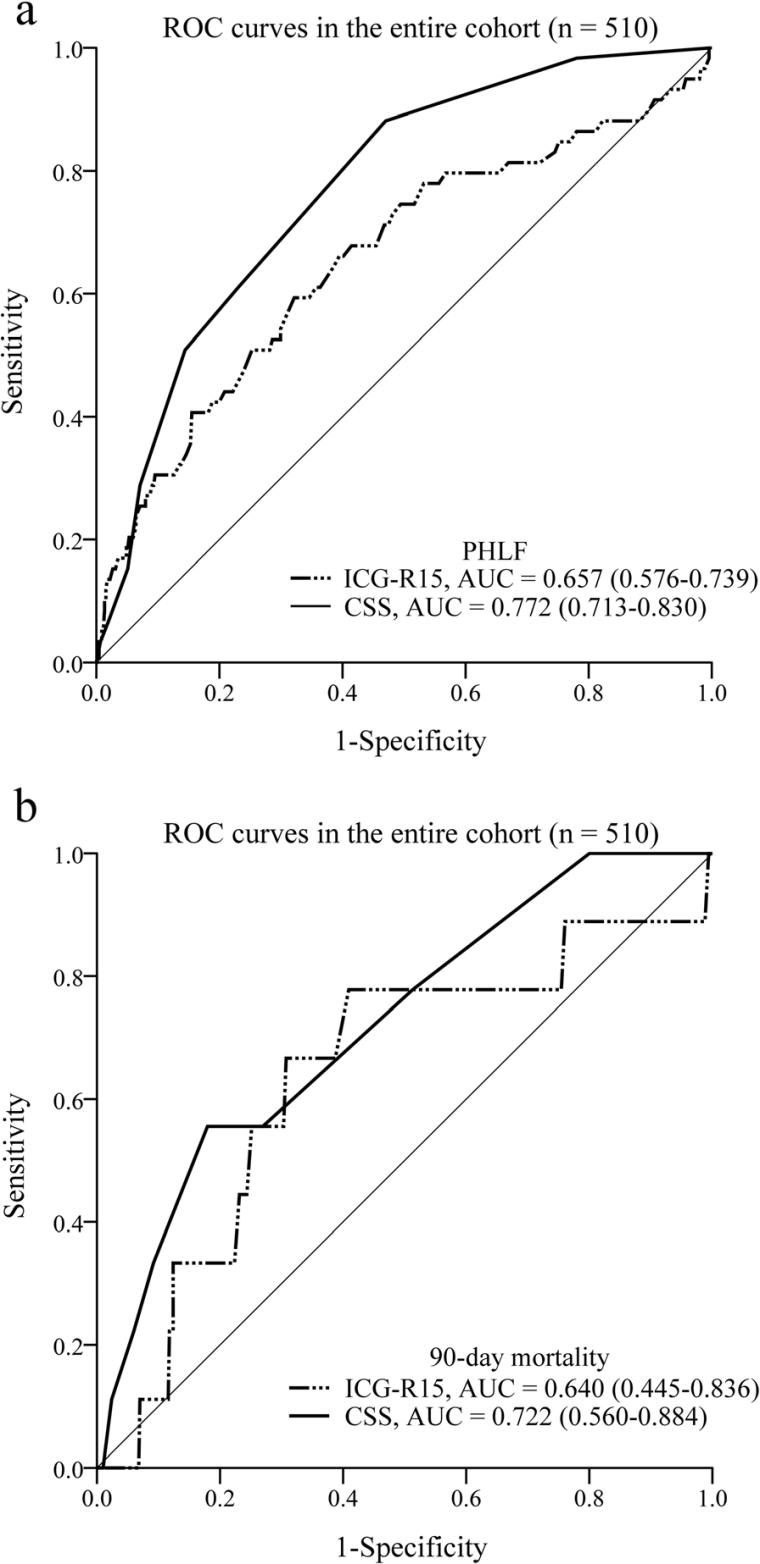
Receiver operating characteristic (ROC) curves and the corresponding areas under the ROC curves (AUCs) of ICG-R15 and CSS in predicting PHLF (**a**) and 90-day mortality (**b**) in the entire cohort. ICG-R15, indocyanine green retention rate at 15 min; CSS, cirrhotic severity scoring; PHLF, posthepatectomy liver failure

A total of 59 patients (11.6%) developed PHLF (Table 2). Using ROC analysis, CSS was found to have a greater ability to predict PHLF compared with ICG-R15 (Fig. 5a). The AUC of CSS in predicting PHLF was 0.772, which was significantly higher than that of ICG-R15 (AUC = 0.657; *P* = 0.004).

The incidence of 90-day mortality was 1.8% (*n* = 9). Eight patients died of PHLF, and the remaining 1 patient died of acute respiratory distress syndrome. The 90-day mortality rate in patients with PHLF was 13.6%, which was significantly higher than that in patients without PHLF (0.2%; *P* < 0.001). ROC analysis showed that CSS was superior to ICG-R15 in predicting 90-day mortality (Fig. 5b). The AUC of CSS in predicting 90-day mortality was 0.722, which was higher than that of ICG-R15 (AUC = 0.640), although the difference was not statistically significant (*P* = 0.379).

## Discussion

ICG-R15 test is a well-established method to assess liver functional reserve. Based on the levels of ICG-R15, a decision tree was proposed for guiding the safe extent of hepatectomy, and ICG-R15 test was regarded as the most determining factor in this decision tree [19]. For example, trisectorectomy or bisectorectomy can be allowed in patients with an ICG-R15 value below 10%. However, this algorithm did not take cirrhotic severity into consideration. The present study indicated that in 446 patients with ICG-R15 in the normal range, the proportion of patients with moderate or severe cirrhosis was as high as 50.7%. Under this circumstance, it would not be appropriate to determine the extent of hepatectomy only based on the ICG-R15 value. Cirrhotic severity cannot be neglected when making surgical decisions. A study conducted by Ishikawa in 139 HCC patients undergoing hepatic resection over a 10-year period revealed that both liver cirrhosis and ICG-R15 had a marked effect on the incidence of postoperative complications [20]. Our previous research also demonstrated that the incidence of PHLF was 38.1% in patients with moderate cirrhosis after resection of 3 or more liver segments and 63.2% in those with severe cirrhosis after resection of 2 or more liver segments. Thus, only minor resection could be recommended to patients with moderate or severe cirrhosis [6].

Recently, Moller and colleagues have evaluated the retention rate of ICG in both healthy subjects and cirrhotic patients with different Child-Pugh grades and found that ICG-R15 was higher in cirrhotic patients than in healthy subjects and increased with the increment of Child-Pugh grades [21]. Lau et al. [22] also found that ICG-R15 in HCC patients with cirrhosis or chronic active hepatitis was higher than that in patients with normal liver. It was suggested that intrahepatic vascular shunts and sinusoidal capillarization might be the main reasons why ICG-R15 was increased in patients with cirrhosis [11]. However, whether ICG-R15 is helpful in predicting the pathological severity of liver cirrhosis in HCC patients with Child-Pugh grade A liver function remains unknown. To the best of our knowledge, the present study was the first in this regard. In this study, liver cirrhosis was pathologically staged according to the Laennec staging system. Although ICG-R15 was increased with more severe liver cirrhosis in HCC patients with Child-Pugh grade A liver function, correlation analysis revealed that ICG-R15 was weakly correlated with the Laennec stage (*r* = 0.325; *P* < 0.001). Besides, the majority of patients in the corresponding cirrhotic stage had an ICG-R15 value below 10%. ROC analysis further showed that the AUCs of ICG-R15 in the diagnosis of moderate and severe cirrhosis were only 0.663 and 0.707, respectively. These results indicated that ICG-R15 was not reliable in assessing cirrhotic severity in HCC patients with Child-Pugh grade A liver function.

In recent years, several non-invasive methods based on serologic tests and imaging techniques have been developed to evaluate liver fibrosis, including aspartate aminotransferase-platelet ratio index (APRI) [23], fibrosis index based on the four factors (FIB-4) [24], King’s score [25], and transient elastography (TE, FibroScan) [26]. However, these methods were employed to assess the severity of liver fibrosis rather than cirrhosis. It is unclear whether they are effective in evaluating cirrhotic severity in HCC patients. In 2016, we proposed a non-invasive method named CSS to predict cirrhotic severity [14]. The CSS is based on clinical parameters that are easily obtained through preoperative examinations. In this study, we demonstrated that CSS was significantly correlated with the pathological severity of liver cirrhosis in HCC patients with Child-Pugh grade A liver function (*r* = 0.788; *P* < 0.001), consistent with previous findings [14]. In addition, we compared the efficacy of ICG-R15 with CSS in assessing cirrhotic severity and found that CSS showed a stronger correlation with the pathological severity of liver cirrhosis than did ICG-R15 and a better performance than ICG-R15 in the prediction of moderate and severe cirrhosis. Our study further revealed that in patients with a normal ICG-R15 value, the accuracy of CSS in diagnosing no/mild, moderate, and severe cirrhosis was 89.09%, 72.78%, and 72.06%, respectively. Therefore, CSS could function as a useful non-invasive method to assess cirrhotic severity even in HCC patients with an ICG-R15 value below 10%.

PHLF is a dreadful complication after hepatectomy and remains the main cause of perioperative death [4–6]. In the present study, 11.6% of patients developed PHLF. The 90-day mortality rate in patients with PHLF was up to 13.6%, whereas that in patients without PHLF was only 0.2%. ROC analysis revealed that the AUCs of CSS in predicting PHLF and 90-day mortality were higher than those of ICG-R15, indicating that CSS had a better predictive value for PHLF and 90-day mortality than ICG-R15.

Several limitations exist in this study. First, this was a retrospective study, thus selection bias could occur. Second, the majority of HCC patients in this study were infected by hepatitis B virus, which is different from patients infected by hepatitis C virus in most Western countries or Japan. Third, our study took place in a single-center, and only patients with Child-Pugh grade A liver function were included in this study. Thus, further larger and multicenter studies including patients with different Child-Pugh grades are needed to validate the results of the current study.

## Conclusions

There was only a mild relationship between ICG-R15 and the pathological severity of liver cirrhosis in HCC with Child-Pugh grade A liver function, and the majority of patients in various degrees of liver cirrhosis had a normal ICG-R15 value. CSS was more effective than ICG-R15 in evaluating cirrhotic severity and still functioned well in patients with ICG-R15 in the normal range. In addition, CSS was superior to ICG-R15 in predicting PHLF and 90-day mortality. More studies are needed to further validate CSS in patients with different Child-Pugh grades.

## Data Availability

The datasets used and analyzed during the current study are available from the corresponding author on reasonable request.

## Abbreviations

HCC: Hepatocellular carcinoma
CSS: Cirrhotic severity scoring
ICG-R15: Indocyanine green retention rate at 15 min
PHLF: Posthepatectomy liver failure
ROC: Receiver operating characteristic
AUC: Area under the ROC curve
APRI: Aspartate aminotransferase-platelet ratio index
FIB-4: Fibrosis index based on the four factors
TE: Transient elastography
H&E: Hematoxylin-eosin
ALT: Alanine aminotransferase
AST: Aspartate aminotransferase
INR: International normalized ratio
PT: Prothrombin time
RBC: Red blood cell
